# Implementation of a Sequence-to-Sequence Stacked Sparse Long Short-Term Memory Autoencoder for Anomaly Detection on Multivariate Timeseries Data of Industrial Blower Ball Bearing Units

**DOI:** 10.3390/s23146502

**Published:** 2023-07-18

**Authors:** Elisavet Karapalidou, Nikolaos Alexandris, Efstathios Antoniou, Stavros Vologiannidis, John Kalomiros, Dimitrios Varsamis

**Affiliations:** 1 Department of Computer, Informatics and Telecommunications Engineering, International Hellenic University, 62124 Serres, Greece; eliskara3@ihu.gr (E.K.); nikosalexandris1990@gmail.com (N.A.); ikalom@ihu.gr (J.K.); dvarsam@ihu.gr (D.V.); 2 Department of Informatics and Electronics Engineering, International Hellenic University, 57400 Thessaloniki, Greece; antoniou@ihu.gr

**Keywords:** anomaly detection, autoencoder, deep learning, industrial data, long short-term memory, predictive maintenance

## Abstract

The advent of Industry 4.0 introduced new ways for businesses to evolve by implementing maintenance policies leading to advancements in terms of productivity, efficiency, and financial performance. In line with the growing emphasis on sustainability, industries implement predictive techniques based on Artificial Intelligence for the purpose of mitigating machine and equipment failures by predicting anomalies during their production process. In this work, a new dataset that was made publicly available, collected from an industrial blower, is presented, analyzed and modeled using a Sequence-to-Sequence Stacked Sparse Long Short-Term Memory Autoencoder. Specifically the right and left mounted ball bearing units were measured during several months of normal operational condition as well as during an encumbered operational state. An anomaly detection model was developed for the purpose of analyzing the operational behavior of the two bearing units. A stacked sparse Long Short-Term Memory Autoencoder was successfully trained on the data obtained from the left unit under normal operating conditions, learning the underlying patterns and statistical connections of the data. The model was evaluated by means of the Mean Squared Error using data from the unit’s encumbered state, as well as using data collected from the right unit. The model performed satisfactorily throughout its evaluation on all collected datasets. Also, the model proved its capability for generalization along with adaptability on assessing the behavior of equipment similar to the one it was trained on.

## 1. Introduction

The continuous technological advancement of the industrial sector, which now is going through its fourth industrial revolution, widely recognized as Industry 4.0 (I4.0) [1], has paved the way for the development and deployment of modern, reliable and robust systems and protocols regarding business sustainability [2]. Industries are always trying to stay relevant to market demands while also establishing a competitive presence in their respective fields. Therefore, one of their principal objectives is to achieve economic profit via cost efficiency throughout the production process. The prevailing course of action to mitigate production costs is to ensure the healthy, practical and effective performance of the manufacturing machinery through a maintenance policy, since defective equipment may lead to catastrophic failures and have a negative financial impact on enterprises.

### 1.1. Maintenance Policies

Traditional maintenance strategies include Corrective Maintenance (CM), Preventive Maintenance (PvM) and Condition-based Maintenance (CbM) [3]. Although a Run-to-Failure (R2F) approach is considered one of the main strategies, it is contradictory to the general opinion of keeping a good equipment performance, since Corrective Maintenance is an unplanned reactive technique that relies on repairs only after any asset failure occurs. This is a high-risk policy due to its unexpected circumstances regarding equipment downtime and extent of damage that can lead to major expenses and hinder production for an unspecified period. On the contrary, Preventive Maintenance is a policy that relies on scheduling, and any maintenance tasks such as machinery inspections, repairs, rebuilds or adjustments follow a time-sensitive plan of action [4]. Despite the fact that this strategy has a higher probability of failure mediation, it occasionally leads to redundant activities, further adding to production costs or to the premature disposal of assets with a promising Remaining Useful Life (RUL). Finally, the digital revolution as well as the technological advances of Industry 4.0 have led to Condition-based Maintenance, a strategy that depends on the continuous observation of the equipment’s mechanical condition and health status with the help of tools set in place to obtain measurements through vibration monitoring, thermography, ultrasound, infrared imaging, tribology, visual inspection, etc. [5]. This is another unplanned method, and any activity by the maintenance management regarding industry machinery is based on the regular analysis of the monitoring data by experienced consultants and the quick actions performed upon detection of anomalies in data readings.

#### Predictive Maintenance

While the constant monitoring using human resources may present a good way of preventing failures and performance issues, it is not the most productive and cost-effective course of action, since the improvements in the industrial sector, particularly the Industry 4.0 innovative technologies [6], allowed the emergence of a more beneficial maintenance policy, the widely known Predictive Maintenance (PdM) [7,8]. Although the term was used to describe Condition-based Maintenance practices twenty years ago [9], it currently is recognized for its utilization of Industry 4.0 technologies, mainly the Industrial Internet of Things (IIoT), Artificial Intelligence (AI), sensors, Big Data and the Cloud. Modern digitization and automation systems set in place by industries that follow smart manufacturing resulted in a great amount of data accumulation, further reinforcing the fundamental purpose of a Predictive Maintenance strategy, which is to allow the scheduling of maintenance actions before any damaging consequences occur to the manufacturing process by identifying an imminent asset failure or an anomalous equipment behavior through inference that is based on collected historical operating data [10].

In general, after the data collection and the necessary preprocessing, a suitable model with a specific architecture is developed for the purpose of training on the aggregated data and learning the underlying patterns that characterize the data. Such endeavor would be difficult, time-consuming or even impossible to identify by the normal means of statistical analysis and condition monitoring due to possible data complexity. Therefore, a major component of a present-day Predictive Maintenance strategy is the use of Artificial Intelligence and especially its subfields of Machine Learning (ML) and Deep Learning (DL) that enable the development of algorithms and models [11] that have the ability to deconstruct the data and recognize the hidden mathematical rules they abide by. Undoubtedly, this procedure demands numerous calculations, and as the complexity of the model increases, so does the amount of the arithmetic parameters that need to be calibrated during the training step. The growing presence of ML applications and their integration into maintenance policies [12] is both due to the increasing computing power of current processors, allowing the deployment of more intricate model architectures and the advances of the scientific field through innovative research initiatives.

### 1.2. Data Availability

Evidently, data availability is an imperative component to a Predictive Maintenance policy utilizing Machine Learning techniques, and although it appears to be an undemanding task due to I4.0 technologies, the acquisition of real industrial operational datasets can prove challenging, particularly to businesses implementing such policies for the first time. Oftentimes, researchers resort to simulated or synthetically generated data to test their scientific approaches [13,14], seeing that real-case records for specific operating conditions are difficult to come by [15].

It is worth noting that healthy production line data are comparatively easier to obtain, since fault conditions are less frequent in regard to their occurrence and documentation; thus, simulated datasets can present a way to train an ML model on exact labeled error conditions or to evaluate an already trained ML model, specifically in the case of anomaly detection where only normal operation data are available [16]. Furthermore, the acquisition of annotated datasets is especially challenging due to the expenses and time needed for the sensitive task of labeling, since experienced human labor must be involved, and the businesses that have carried through these processes understandably need to keep their data confidential [17].

The present work attempts the development and training of a Deep Learning model for the case of anomaly detection in a time series using real multivariate temporal data collected from actual operating production plant equipment via sensor devices, thus avoiding any concerns regarding synthetic data and their statistical representation and logical validation [18]. More information about the data in this work is presented in Section 2 of this article. All data were made publicly available via the Zenodo platform in https://doi.org/10.5281/zenodo.7994124, accessed on 1 June 2023.

### 1.3. Model Overview

This work attempts the development of a Deep Learning temporal anomaly detection model [19], specifically based on the Autoencoder (AE) architecture [20,21] and the utilization of Long Short-Term Memory (LSTM) cells. In comparison to traditional methods, the use of LSTMs in predictive maintenance provides notable advantages by leveraging the power of recurrent neural networks, enabling the automatic extraction of relevant features and eliminating the need for manual feature engineering, which can be time-consuming and prone to human biases. This study introduces a novel dataset obtained from an industrial blower. The dataset encompasses measurements from both the right and left mounted ball bearing units, collected over several months under typical operational conditions, as well as during a encumbered operational state. The primary focus of this study is to develop an anomaly detection model that could effectively examine the operational behavior of the two bearing units. Specifically, we successfully trained a stacked sparse LSTM Autoencoder on the data gathered from the left unit during normal operating conditions. This model was able to capture the underlying patterns and statistical relationships present in the data allowing it to distinguish between the two operational states of the system.

**Figure 1:**
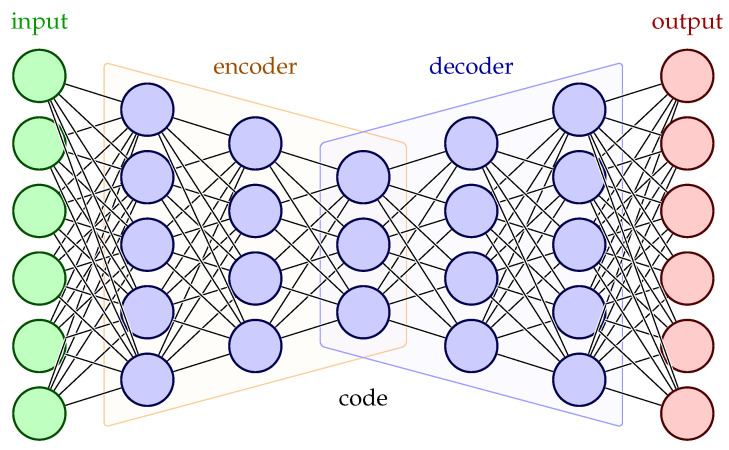
Typical Autoencoder Architecture.

Autoencoders are composed of an encoder and a decoder. The inputs proceed through the encoder, are deconstructed to latent representations, and then the decoder tries to reconstruct those encodings as close as possible to the given inputs. The evaluation of this method relies on the reconstruction error of the described technique. Figure 1 presents a straightforward visualization of a simple Autoencoder architecture. The LSTM cell constitutes an evolution of the simple Recurrent Neural Network (RNN) cell which can lead to difficulties on the subject of unstable gradients (vanishing or exploding) and short-term memory in the DL model’s effort to identify patterns in terms of data periodicity [22]. Consequently, the LSTM cell is the main building block of our AE model for its ability to process sequential data and retain or discard any input information that may adversely affect its output by maintaining a memory state. Additionally, due to the limited number of input variables in the data, a regularization term was implemented to the latent data representations for the purpose of adding a sparsity penalty to the coding parameters and ensuring the most important features are preserved. All of these concepts are explained in further detail in Section 2 of this article.

### 1.4. Related Works

The Deep Learning task of anomaly detection via an Autoencoder (AE) architecture relies on the AE model’s training on normal production data and learning to reconstruct it with minimal reconstruction error, so any data reconstruction exceeding a predefined acceptable error is considered an anomaly. Apart from AEs, other main Machine Learning and Artificial Neural Network (ANN) methods were presented by T.P. Carvalho et al. [23] in a systematic literature review in the frames of Predictive Maintenance regarding industrial equipment. It also lists four public datasets to support the testing and assessment of various PdM methodologies. Additionally, a PdM systematic literature review recently carried out by O.Ö. Ersöz et al. is available in [24], aiming specifically for the field of transportation systems.

A detailed survey was also conducted by B. Lindemann et al. [25] focusing on state-of-the-art anomaly detection techniques using LSTM networks. Besides LSTM-only methods, Autoencoders utilizing LSTMs were highlighted for their significance in unsupervised applications, as well as other hybrid architectures. Recent trends of graph-based and transfer learning approaches were also presented. An unsupervised graph-based approach was introduced by E.S. Miele et al. [26] that combined a Graph Convolution Network (GCN) with Autoencoder architecture for anomaly detection in a multivariate time series. The model of this work was trained on wind turbine data collected by a Supervisory Control and Data Acquisition (SCADA) monitoring system, and for its evaluation, a four-stage threshold methodology was implemented to allow warnings only for significant reconstruction errors. The framework showed great performance by detecting anomalies before any SCADA alarms and was compared to other promising applications on wind turbine data, an LSTM-AE and an attention-based Convolutional Neural Network–Long Short-Term Memory (CNN-LSTM) model [27,28].

Three different AE architectures were developed by M. I. Radaideh et al. [29] utilizing bi-directorial LSTM units, bi-directorial Gated Recurrent Units (GRUs) and a convolutional LSTM (convLSTM) approach for univariate time-series anomaly detection. The three models trained on real experimental waveform signals from a subsystem of a high-voltage converter modulator [30], a critical power system at the Spallation Neutron Source and tested on never-seen-before normal and faulty pulses generated by the subsystem. All Autoencoders achieved the desired outcome, meaning a higher reconstruction error while performing evaluation of abnormal pulses, with a similar performance among all three. Additional anomaly detection methods were applied on the training data including Isolation Forest (IF), Support Vector Machine (SVM), Local Outlier Factor (LOF), Random Forests (RF), Decision Trees (DT), K-Nearest Neighbors (KNN), Convolutional Neural Network (CNN), CNN Autoencoder (CNN-AE) and Feed-Forward Neural Network Autoencoder (FFNN-AE). In terms of comparison, the three proposed AEs achieved an average of 0.89 Area Under the Curve (AUC) score, highly prevalent over the 0.68 average of all the other methods. It is worth noting that all AE techniques had the longest training computing times.

A novel adaptable predictive maintenance framework was introduced by G. Hajgató et al. [31] that incorporated a Deep Convolutional Autoencoder (DCAE), a clustering algorithm [32] and a Principal Component Analysis (PCA) in a case study regarding oil degradation of an industrial gearbox. Firstly, the DCAE model was trained on multivariate time-series data of the gearbox in an unsupervised and undercomplete manner; then, its decoder was removed and the test data were encoded to their latent representations. This practice makes use of a common AE application regarding non-linear dimensionality reduction and feature extraction. Afterwards, the encodings were divided into clusters by a K-Means algorithm and the data were once again compressed by PCA and studied in an explainable manner by the use of visualization techniques.

A novel DL methodology was assessed in [33] based on an LSTM Autoencoder architecture for the purpose of fault detection and RUL estimation in the frames of a real-world predictive maintenance application. The study investigated a comparison between predictive and preventive maintenance strategies by following a DL approach regarding the condition of a hot rolling milling machine through historical maintenance records. Three LSTM Autoencoder models were developed, each trained on Temperature and Energy features corresponding to the different conditions of the machine, mainly a good, a bad and an intermediate state. A preliminary evaluation of the AE models on data of every operating condition yielded favorable results in terms of accuracy, successfully recognizing each machine condition.

In the field of medicine, T. Dou et al. [34] examined the Predictive Maintenance application of anomaly detection on proton Pencil Beam Scanning (PBS) delivery systems. LSTM-based Stacked Autoencoder (LSTM-SAE) models with a dropout rate [35] were trained and evaluated on temporal beam data including the most essential system operating parameters. Although system performance is monitored through daily Quality Assurance (QA) before every patient treatment and Preventive Maintenance methods, Predictive Maintenance could be incorporated providing alarms prematurely, thus allowing maintenance actions or inspections in cases of underlying system issues. The models, in terms of identifying anomalous events, were evaluated by the Mahalanobis distance metric, and aside some skew data distribution, the proposed methods performed satisfactorily on normal and abnormal beam data.

Another AE architecture for fault detection developed by X. Liang et al. [36] who studied two cases of a working pump in an oil and petrochemical factory where multivariate data of fifteen measurements were collected from a system monitoring the speed, flow and pressure of the pump under two different operating conditions. A time series of normal operating pump data was used for the training of a Sparse Autoencoder (SAE), which differs from a typical AE model by implementing a sparsity penalty to its loss function for the encodings. Another PCA model was also trained for comparison purposes. The SAE method performed better than the PCA method regarding Receiver Operating Characteristic (ROC) curve and average Area Under the Curve (AUC) values, as well as in fault isolation in terms of a variable mapping indicative of each variable’s contribution to the detected anomaly. Specifically in the first case, a misalignment fault was detected four days earlier by the SAE model and the cause was significantly more evident in the variable mapping, finding that that an increase in temperature for four bearings contributed to the fault.

Our approach is inspired by AE-based architectures for predictive maintenance described above. Following the general strategy of employing state-of-the-art anomaly detection techniques using LSTM networks presented in [25], an Autoencoder was trained using data from a particular set of measurements of the monitored equipment to model the normal operation state of the system. The trained model was in turn used to predict anomalies in the behavior, and hence possible malfunction, of the machine by comparing the actual measurements to the estimated outcomes of the AE. However, it should be made clear that a direct comparison between the approaches available in the literature and our implementation is not possible. The implementation of the AE was tailored on the particular set of measurements produced by the machinery equipment of the experiment operated by our industrial partner in their actual production line. In this sense, it would be very difficult or even irrelevant to try to use some of the aforementioned approaches as out-of-the-box solutions.

The rest of the article is organized as follows: Section 2 presents the industrial machinery and the equipment examined, as well as an overview of the data collected. The theoretical background of the model architecture, the data preprocessing and its training are also described in this section. Section 3 analyzes the results of the evaluation of the model on the various datasets derived from the available data, and Section 4 presents the conclusions and further research thoughts and proposals.

## 2. Materials and Methods

This article presents the development of a sequence-to-sequence sparse stacked Autoencoder model based on LSTM layers and its training through a self-supervised manner for the purpose of detecting anomalous behavior on industrial equipment sensor data. The software stack for the analysis is described in Table 1 and was used in the virtual environment of Google Colaboratory [37].

### 2.1. Factory Machinery

**Figure 2:**
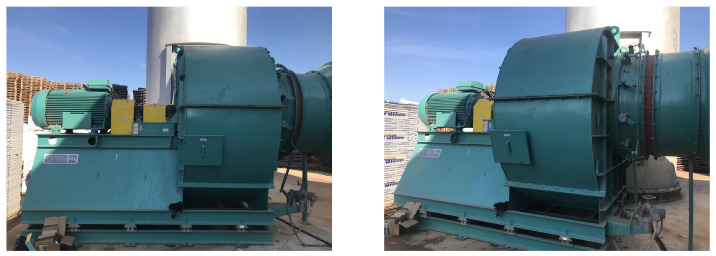
Industrial Blower for temperature regulation via air circulation.

The system investigated in this work is displayed in Figure 2. It is an industrial blower, and its basic operation is the air circulation and supply for cooling purposes and temperature regulation. This specific infrastructure consists of a centrifugal fan, which aims for a uniform and constant air supply by means of a quiet and energy-efficient operation compared to that of an axial fan, and its rotational movement is powered by a three-phase electric motor. Particularly, the working characteristics of fan infrastructure are as follows:

- Nominal Power: 132kW;
- Airflow: $65.7\;m^{3}/s;$
- Pressure: Δp=1600Pa;
- Operation Speed: 980rpm.

The motor is connected to the ventilation system through a rigid shaft on top of which there are two mounted ball bearing units. During the blower’s operation, air enters through the inlet nozzle and exits through the outlet with the aim of removing heat through recirculation. The entire industrial infrastructure is supported by rigid elements, resulting in the production of vibrations. So far, the maintenance plan for the machinery involves performing regular and scheduled maintenance in order to keep it running and prevent any costly unplanned downtime from unexpected equipment failure. Towards the transition from preventive to predictive maintenance policies, sensors were placed on top of the infrastructure’s mounted ball bearing units, mainly the left one closer to the motor, and the right one next to the centrifugal fan according to ISO 20816-03:2022 [38]. The sensor that was used was the BMC0001 condition monitoring sensor from Balluff. The data were received by a Programmable Logic Controller (PLC) and were transported over Message Queuing Telemetry Transport (MQTT) protocol to a cloud server for postprocessing and archiving.

### 2.2. Data Analysis

The available data collected from the industrial system presented above describe the operating behavior of its left and right mounted ball bearing units in terms of velocity, which is expressed in inch/s , and temperature, which is expressed in degrees of Fahrenheit , according to their original raw values. However, a conversion was made to the International System of Units (S.I.) by expressing the velocity in m/sec and the temperature in degrees of Celsius . To clarify, velocity is provided by the metric of V-RMS (Velocity Root Mean Square Error) commonly used in vibration analysis, where the term of velocity is defined as the equipment’s rate of displacement at a given moment during its operation. The calculation of the V-RMS follows the below formula:

(1) $$\mathrm{VRMS}=\sqrt{\frac{1}{n}\cdot \sum_{i=1}^{n}v_{i}^{2}},$$

where:

- *n* is the amount of velocity readings,
-
$v_{i}$ are each one of the velocity readings.

All the velocity readings are captured by the sensor device within a configured time window, so the outputted measurement is an indicative average of the bearings’ operating velocity representing their underlying health condition. The temperature feature and its contribution is self-explanatory.

**Figure 3:**
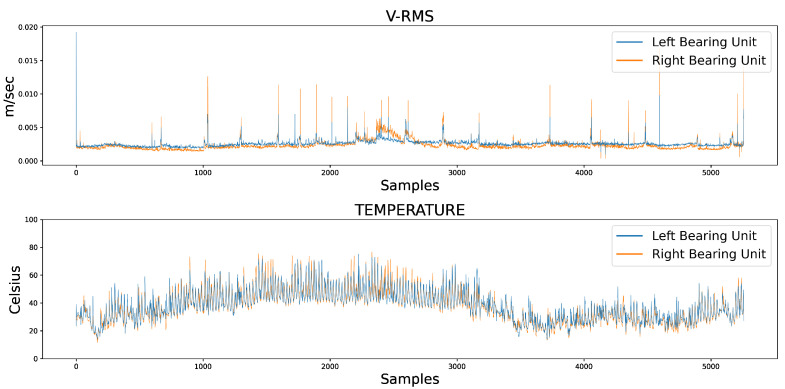
Comparative overview of the V-RMS (**top**) and Temperature (**bottom**) attributes of the left and right ball bearing units during normal operation.

**Figure 4:**
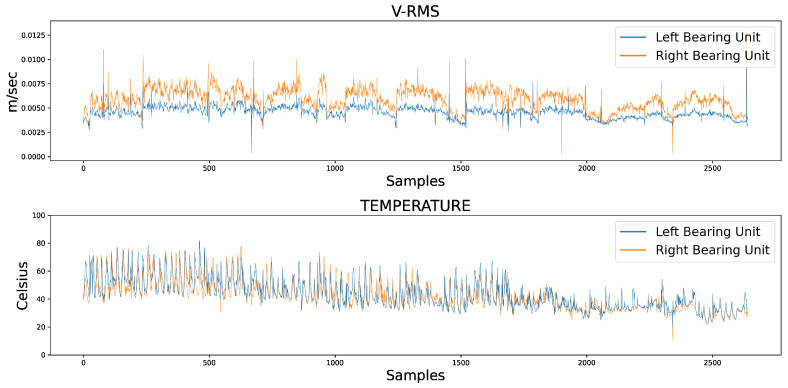
Comparative overview of the V-RMS (**top**) and Temperature (**bottom**) attributes of the left and right ball bearing units during encumbered operation.

Figure 3 presents the V-RMS and Temperature temporal data measurements of both the left and right mounted ball bearing units during normal machinery operation, while Figure 4 shows the same unit attributes during an encumbered operating state later in production.

The samples of the available datasets were captured by sensing devices over a period of several months. Continuous records of a low V-RMS value, indicative of a powered down system at the time of the sensor’s captured reading, were filtered out of the datasets, as well as instances of intentional shutdowns for maintenance purposes. As an interesting remark, several apparent momentary spikes in V-RMS values were a normal operational occurrence, mainly due to unexpected power outages that forced the plant machinery to restart, subsequently causing increased start-up loads producing larger vibration values. Despite the sizeable difference between the spikes and normal values of the time series, their instantaneous behavior was within the framework of a conventional industrial operation and their removal was not mandated so as to not disrupt the data, and the chosen course of action regarding the spikes was to filter the time series through a rolling median, which is presented later in this work. Additionally, comparing the opposite spike instances between the left and right mounted ball bearing units during normal operation in Figure 3, the higher peaks of the right unit were caused by the unit’s proximity to the centrifugal fan of the industrial machinery, which is also the cause for its higher V-RMS values at the encumbered operational state.

At this point, it is worth noting that the encumbered state does not necessarily suggest a fault condition of the machinery, since its overload was not imperative to cause a shutdown due to an equipment defect or failure during the several months of data readings. In the context of this work, the standard operational condition was used as a foundation for the development of an Autoencoder model that was trained to recognize overloaded circumstances, comparable in certain respects to an anomaly detection task. In addition, the training of the AE model was based solely on the left unit’s V-RMS and Temperature readings due to the fact of a slightly burdened state, although not necessarily erroneous, halfway through the right unit’s operation that would most likely affect model training. However, the right unit’s data served as a means of evaluating the model’s performance and adaptability on data of similar equipment structure and working condition.

Finally, a naive comparison between the readings captured during standard and encumbered operation can be realized through each attribute’s statistical mean as depicted in Table 2, and it is apparent that the increased attribute values confirm the execution of a more strenuous ball bearing activity.

### 2.3. Model Development and Training

#### 2.3.1. Machine Learning

**Figure 5:**
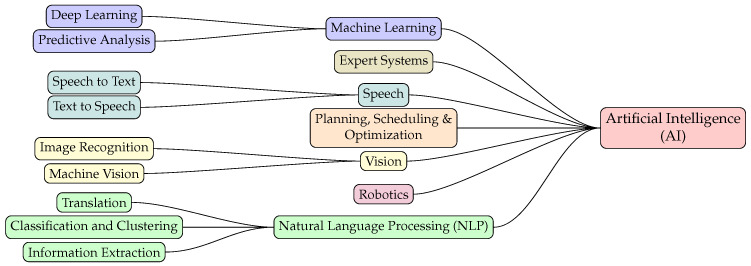
Fields of Artificial Intelligence.

Francois Chollet, the creator of the highly popular Keras library, describes Artificial Intelligence (AI) as “the effort to automate intellectual tasks normally performed by humans” [39]. Since 1950s, the prevalent approach to define human decision making and logical problem solving was made possible through explicit programming rules, but it lacked the capability of recognizing the principles of more complex problems. Today, the field of AI has evolved to include many subfields, an overview of which is shown in Figure 5.

The Machine Learning branch of AI rose to be one of the most popular ones due to the development of algorithms capable of learning from data through training instead of hardcoded instructions. The successful training of an ML system implies that the system under investigation can properly analyze historical data through structured mathematical calculations and identify the statistical rules and hidden patterns that the data follow in such a way as to make adequate predictions when new data are introduced. The inference capabilities of the model, as part of its prediction, depends on the ML application that it is trained for. The rapid progress of ML in the recent years can be attributed both to the increase in dataset availability and in advances in hardware computing power that have allowed the development of ML algorithms; for this work, the ML technique employed is the Artificial Neural Network [40].

An essential component regarding the training process of a Machine Learning model in general as well as a decisive factor to model selection, is the availability in terms of data annotation. An ML model can be trained in a supervised or unsupervised fashion with respect to the presence or absence of target outputs in the dataset, respectively. Apart from these two distinct learning methods, and among others out of the scope of this article, semi-supervised learning allows an ML model to be trained on both labeled and unlabeled instances of a dataset, while a model based on a self-supervised learning method utilizes its inputs to generate targets. In the frame of the present work, a self-supervised approach is utilized for the purpose of training an Autoencoder model.

#### 2.3.2. Long Short-Term Memory

The fundamental high-level structure of an ANN is reflected through its layer organization, while the nature of each network layer is dictated by the functionality of its cells, which can vary depending on the ML task that needs to be accomplished and the data that need to be processed. In the frame of this work, since our data are in the form of time series, the LSTM unit is our chosen cell architecture, and, by extension, the LSTM layer.

**Figure 6:**
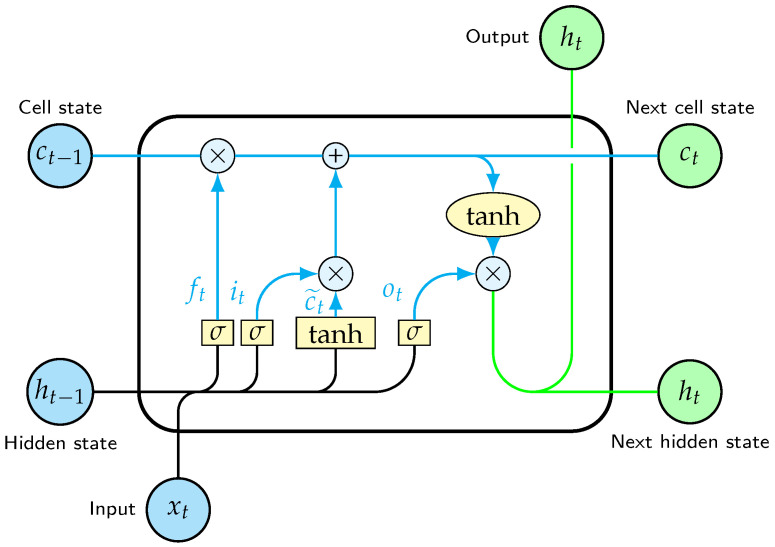
Internal architecture of an LSTM cell.

**Figure 7:**
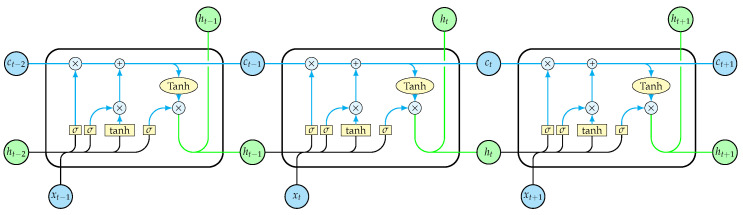
An LSTM cell unfolded through time.

The LSTM architecture belongs to the class of recurrent neural networks. The simple RNN unit is distinguished by its ability to handle sequential data, such as time series, text, audio, etc., where the order of the information matters, by taking into account past dependencies within the sequence. RNN units implement an internal feedback loop that allows the processing of a sequence element through the influence of the processed output of the previous element, thus maintaining a continuous memory state of past information. Despite their capabilities, RNN’s challenge lies to the handling of longer sequences, which causes gradients to vanish or become unstable by exploding as the number of stages they have to go through increases, and the difficulty of maintaining long-term dependencies becomes apparent. The LSTM cell is one baseline variant of the RNN unit that was introduced to address the gradient problem [41]. Figure 6 presents the structure of the LSTM cell, while Figure 7 shows the iterative process within the cell unfolded through time as each sequence element is introduced.

The data are introduced to an LSTM neural network in a sequential manner as three-dimensional inputs in the form of (batch_size, time_steps, features), where batch_size refers to the number of sequences that pass through the network, time_steps is the length of each sequence, which also defines the number of iterations of the internal cell loop, and features is the number of data characteristics to be used in the model training, excluding the labels.

The key components of the LSTM cell are two memory states, namely the long-term memory or carry state *c* and the short-term memory or hidden state *h*, and three memory gates in the form of a sigmoid transformation, namely the forget gate, the input gate and the output gate (from left to right as shown in Figure 6). The LSTM cell consists of four simple fully connected one-layer neural networks, each indicated by its utilization of a bias term, and their outputs for every time step are calculated by the following equations (from left to right as shown in Figure 6):

(2) $$f_{t}=\sigma \left(W_{f}\cdot \left[x_{t},h_{t-1}\right]+b_{f}\right),$$

(3) $$i_{t}=\sigma \left(W_{i}\cdot \left[x_{t},h_{t-1}\right]+b_{i}\right),$$

(4) $${\tilde{c}}_{t}=\tanh\left(W_{\tilde{c}}\cdot \left[x_{t},h_{t-1}\right]+b_{\tilde{c}}\right),$$

(5) $$o_{t}=\sigma \left(W_{o}\cdot \left[x_{t},h_{t-1}\right]+b_{o}\right),$$

where:

- *t* is the current time step, or element, of the input sequence,
-
$f_{t}$ is the forget gate vector,
-
$i_{t}$ is the input gate vector,
-
${\tilde{c}}_{t}$ is the temporary cell input vector,
-
$o_{t}$ is the output gate vector,
-
$x_{t}$ is the current element of the input sequence,
-
$h_{t-1}$ is the previous cell’s output,
-
$W_{k}$ are the weight matrices for every one-layer neural network,
-
$b_{k}$ are the bias vectors for every one-layer neural network,
-
σ is the sigmoid activation function,
-
tanh is the hyperbolic tangent activation function.

Out of the four one-layer simple neural networks of the LSTM cell, the one implementing the tanh activation function can be considered as the equivalent of a typical ANN layer and its activation, thus ${\tilde{c}}_{t}$ acts as the main analyzed input of the cell that is to be considered for the long-term memory. Additionally, the cell’s outputs in time step *t* are calculated as given below:

(6) $$c_{t}=c_{t-1}\cdot f_{t}+{\tilde{c}}_{t}\cdot i_{t},$$

(7) $$h_{t}=\tanh\left(c_{t}\right)\cdot o_{t},$$

where:

-
$c_{t-1}$ is the previous long-term memory,
-
$c_{t}$ is the updated long-term memory,
-
$h_{t}$ is the cell’s main output.

The three gates apply a sigmoid activation function σ to their weighted inputs and bias, so their outputs are scaled values between 0 and 1, which is indicative of irrelevant or relevant information with respect to these boundaries, as their next operation is an element-wise multiplication and these gates are responsible for what information should be preserved and what should be discarded.

Specifically, the forget gate $f_{t}$ controls the long-term memory state up to the previous time step $c_{t-1}$ and what elements should be erased, while the input gate $i_{t}$ controls the current temporary cell input ${\tilde{c}}_{t}$ and what new information from time step *t* should be added to the long-term memory $c_{t}$ through Equation (6). The output gate $o_{t}$ controls the long-term memory state up to the current time step $c_{t}$ , as shown in Equation (7), and what information should be extracted to act as the short-term memory $h_{t}$ and cell output. All gates take into account information from the current sequence element $x_{t}$ and the previous cell output $h_{t-1}$ , and gate values closer to 0 denote irrelevant cell memories to be erased, while values closer to 1 allow cell memories to be kept as relevant.

Another important distinction to be made regarding the LSTM layer is its output. As briefly stated, an LSTM layer accepts three-dimensional inputs (batch_size, time_steps, features), meaning that each sequence of the batch that is being processed through the layer is a two-dimensional array of shape (time_steps, features). An LSTM layer of size *s* can output another two-dimensional array of shape (time_steps, s), where each sequence element is each cell output $h_{i}$ for every time step $i=1,\ldots ,{\mathrm{time}}_{-}\mathrm{steps}$ and *s* is the new altered feature space, or it can output a vector of size *s*, which corresponds only to the cell output of the last time step. The LSTM output depends on the chosen model architecture and its structure requirements.

#### 2.3.3. Autoencoder

An Autoencoder is a neural network model that depends on reconstructions. It consists of an encoder that transforms the model’s inputs to codings representative of the most essential underlying data patterns and a decoder that is tasked to convert those codings to outputs as close as possible to the original inputs as depicted below: 
(8)

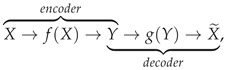


where:

- *X* is the input to the AE,
- *Y* is the coding,
-
$\tilde{X}$ is the reconstructed output,
- *f* is the encoder layer transformations,
- *g* is the decoder layer transformations.

The successful training of the AE is accompanied by the minimization of a loss function that measures the error between the original input data *X* and the model reconstructions $\tilde{X}$ . To simplify, an AE learns to copy its inputs to its outputs, although the triviality of an AE acting as an identity function is not the desired objective and it can be circumvented by implementing certain restrictions throughout the AE’s architecture.

Notably, the dimensionality of the coding space is of great importance, resulting in either an undercomplete or an overcomplete AE model [42]. An undercomplete model has a coding space of lower dimension compared to that of the input feature space, forcing the model to learn latent feature representations through dimensionality reduction, while an overcomplete model has a coding space of equal or higher dimension compared to the input feature space. Despite the fact that an overcomplete model has a higher risk of copying its inputs without learning anything of importance concerning the data, it can be regulated by adjusting its capacity regarding the number of encoder and decoder layers, thus allowing more complex data patterns to possibly be recognized, and, as depicted in Figure 1, a stacked Autoencoder is typically realized in a symmetrical manner. Furthermore, an additional constraint to the overcomplete model can be implemented by adding an appropriate penalty to the model’s loss function, commonly by imposing restrictions to its coding layer through a regularizer applied to the layer’s output, encouraging the model to learn sparse representations of the input data, meaning that the model reduces its available feature space to keep the most relevant space for its coding.

In terms of anomaly detection, the validity of a trained AE model relies on its reconstruction error. An AE model that is trained on data of a particular behavior and pattern returns a small reconstruction error when introduced to never-seen-before data of similar behavior. On the contrary, when the trained AE model is tasked with reconstructing data that are significantly divergent from what it has seen during training, the reconstruction error is notably higher, thus indicating an anomaly condition.

#### 2.3.4. Data Preprocessing

As stated in Section 2.2 of this article, the developed AE model is trained to recognize the behavior that is presented at the standard operational state of the left mounted ball bearing unit, but before the data can be fed into the model and utilized for training as well as validation, they must be preprocessed accordingly.

**Figure 8:**
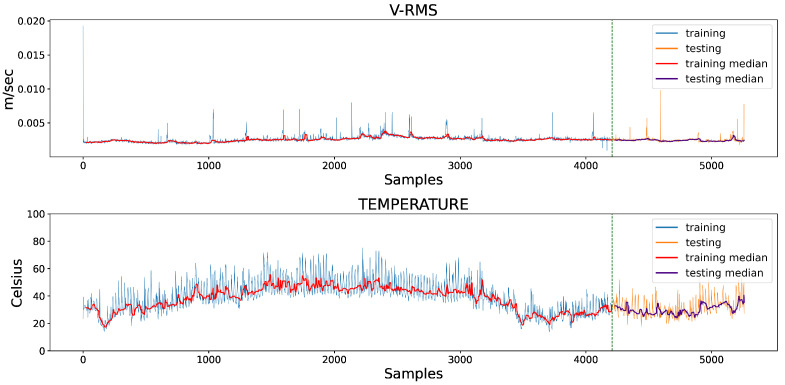
Segmented training and testing datasets of the left mounted ball bearing unit’s V-RMS and Temperature attribute values, as well as the respective filtered values of a rolling median with a moving window of 24 samples.

As a first order of business, out of the 5260 samples of the left unit, the first 80% were used for training, while the remaining 20% served as a testing dataset to evaluate model performance and generalization on records of the same behavior; thus, 4208 observations acted as the training set, while 1052 acted as the testing set. Furthermore, concerning the time-series spikes, the attribute values were filtered through a rolling median, consequently eliminating any imbalances during operation since the median can generally smooth out fluctuations in production data regardless of extreme values. The fixed amount of 24 samples was experimentally selected as the size of the moving window regarding the calculation of the rolling median, which clearly produced reduced datasets of 4185 and 1029 records for the training and testing set, respectively. In Figure 8, an overview of the train/test split can be seen, along with the substitute median values.

Afterwards, the V-RMS and Temperature features of the new values were transformed through a normalization method as both attributes were shifted and scaled to a range with a minimum of 0 and a maximum of 1, following the transformation below:

(9) $$X_{scaled}=\frac{X-X_{min}}{X_{max}-X_{min}}\cdot \left(max-min\right)+min,$$

where:

- *X* represents each feature’s values,
-
$X_{min}$ is the feature’s minimum value,
-
$X_{max}$ is the feature’s maximum value,
-
min is the minimun of the new feature range,
-
max is the maximum of the new feature range,
-
$X_{scaled}$ represents the new normalized feature values.

As a note, the min and max parameters of the formula, which denote the borders of the desired feature range, in this occasion are 0 and 1, respectively, and could have been omitted in Equation (9). The numerical disparity between V-RMS and Temperature would likely lead to unequal and biased attribute treatment by the model during training in terms of mathematical calculations, layer parameter configuring or gradient optimization, resulting in inaccurate model performance and predictions. Feature scaling helps mitigate these consequences caused by imbalanced numerical attributes by constraining values in a homogeneous manner and is a recommended practice for the development of stable and robust DL models. As an important clarification, all datasets used in this work were transformed after the scaler was fitted to the feature values of the training set, preserving the training feature $X_{min}$ and $X_{max}$ values for every subsequent transformation.

In this section, the significance of utilizing LSTM layers in machine learning for training models capable of analyzing sequential data and recognizing dependencies between the sequences is explained in detail, so the final step of the current data preparation is the reshaping of the two-dimensional training dataset to inputs of three dimensions. We recall that the current shape of the training set is $\left(4185,2\right),$ where 4185 is the number of observations and 2 is the number of variables (V-RMS and Temperature), while the input to an LSTM layer is in the form of (batch_size, time_steps, features). A satisfactory course of action is achieved by producing overlapping windows of 24 consecutive observations, resulting in a final three-dimensional training set of shape $\left(4162,24,2\right)$ , consisting of 4162 sequences of 24 successive records; each sequence element is represented by its two attribute values. The same sequential transformation applies to every dataset presented in this work.

#### 2.3.5. Modeling and Training

Table 3 presents the final architecture of the Autoencoder model, which was determined empirically after extensive hyper-parameter experimentation, as well as the dimensionality of each sample as it passes through AE’s layers during training.

The symmetrical structure, with respect to the feature space of the AE, has a configuration of 8-4-2-4-8, where 2 is the feature size of the coding space that holds the latent representations of the input training data, and since it matches the input feature size, the model is considered an overcomplete AE. In addition, the AE model falls within the range of stacked AEs, since the amount of encoder and decoder layers is more than one. The capacity of the AE, along with the increased feature space of its layers, allows potentially more complex patterns to be identified by the model. However, the aforementioned risk of it being incapable of learning anything of importance and acting as an identity function is mitigated by the use of a regularizer term in its coding layer.

Furthermore, the first LSTM encoder layer of the proposed AE architecture accepts the original two-feature input sequences and outputs sequences of an increased feature space of size 8, which in turn are fed to the next LSTM layer that outputs sequences of four features. The last LSTM layer of the encoder accepts the previous layers’ outputted sequences and, after processing the data through its cells, produces only the output of its last cell, meaning the last time step, which is a two-feature vector that represents the compressed original input data. The RepeatVector is simply a bridging layer between the encoder and the decoder, not adding to the model’s parameters, that replicates the encoder’s compressed representation vector 24 times to form once again a sequence, since the next layer of the decoder is an LSTM that requires a two-dimensional array as input. Therefore, the first LSTM layer of the decoder accepts sequences of two features and initiates the reverse process of unraveling the feature space by outputting sequences of four features, which in turn are fed to the decoder’s second LSTM layer that outputs sequences of eight features. Finally, the TimeDistributed (Dense) layer of the AE applies a fully connected linear layer of a two-feature vector output to every time step of the sequence, meaning to every eight-feature sequence element, and produces the final model output sequence of the model, which has a feature space of 2, such as the original inputs.

The loss function which the model would attempt to minimize was chosen to be the Mean Squared Error (MSE) and is computed by the following formula:

(10) $$\mathrm{MSE}=\frac{1}{n}\cdot \sum_{i=1}^{n}{\left(y-\hat{y}\right)}^{2},$$

where:

- *y* represents the original inputs,
-
$\hat{y}$ represents the model’s outputted reconstructions,
- *n* is the number of the given samples.

Since the current model’s inputs are batches of sequences of two features, the final error value is calculated over the mean of each last dimension. Additionally, the loss function includes a regularization term due to the L1 regularization added to the last LSTM layer of the encoder for the purpose of penalizing its parameters, consequently modifying the loss function MSE (10) that the model aims to minimize to the following LF (Loss Function):

(11) $$\mathrm{LF}=\frac{1}{n}\cdot \sum_{i=1}^{n}{\left(y-\hat{y}\right)}^{2}+\lambda \cdot \sum_{i=1}^{m}\left|z_{i}\right|,$$

where:

-
λ is the regularization parameter,
- *m* is the number of parameters in the layer,
-
$z_{i}$ are the values of the layer’s parameters.

During each training step, the gradients of the loss function with respect to the model’s parameters are computed; therefore, the regularized layer’s parameters are impacted by the added penalty term and the model is encouraged to minimize those that contributed more to the error, forcing them closer to zero, thus leading to sparse parameters in the model where those of higher value are indicative of more important features, which is the desired objective of the Autoencoder’s coding layer. In addition, regularization parameter λ is set to 0.00015 , as presented in Table 3, while the optimization method selected for training the model, regarding its parameter reconfiguration throughout the training process, after their respective error contribution is computed, is the Adam optimizer with a learning rate of 0.001 . Both the loss function and the optimizer of the model were chosen through experimental investigation and by monitoring the training progress.

**Figure 9:**
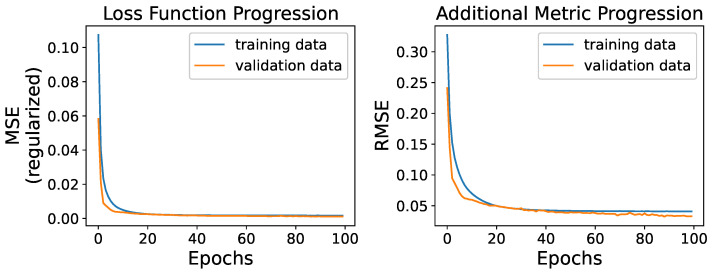
Regularized MSE Loss Function (**left**) and RMSE Metric (**right**) value progress during model training.

After the necessary data preparation and model finalization, the 4162 sequences were shuffled before they passed through the layers of the model for the training process in random batches of 32 instances. A total of 100 epochs was selected as the number of training iterations, and an additional 20% of the training data were randomly reserved for validation and monitoring model performance during training throughout the epochs which the model did not train on. Moreover, the metric of Root Mean Squared Error (RMSE) was also observed throughout the training process as additional useful information for model evaluation, along with the values of the modified loss function represented by the average across all the batch computed losses in each epoch. The monitored loss function and metric values over the 100 epochs are presented in Figure 9.

The training of the model throughout the epochs seems to be successful since it performs satisfactorily on both the training and the reserved never-seen-before validation data. The regularized loss function appears to minimize successfully, while the model’s performance on the validation data, in terms of loss and metric values, follows the pattern of training data without any significant deviation that would have the validation values increasing. Specifically the final epoch yielded an average loss of 0.001712 and an RMSE of 0.040854 on the training data, while the respective values on the validation data were 0.001125 and 0.033024 . The training progress is indicative of a well-trained model that would generalize well on new instances that follow similar data behavior, and, as an important remark, its evaluation will be validated through the means of the unregularized MSE loss function of Equation (10).

## 3. Results

### 3.1. Evaluation Threshold

As mentioned, an AE trained model for anomaly detection purposes is evaluated on its reconstruction error of the inputs it processes, and, if that error exceeds a predetermined threshold, then an anomaly condition is captured indicating dissimilarity to the data on which the AE model was trained. This work follows a naive threshold approach since the encumbered operational state of the two mounted ball bearing units was not erroneous for the industrial machinery, and the term anomaly refers mainly to the bearings’ diverging reading behavior rather than a fault condition being detected. In that regard, the trained AE model was used for inference on the entire training set of 4162 sequences as described in Section 2.3.4, including the reserved validation data that were not utilized in the training process, and the reconstructed sequences were obtained. Finally, the MSE loss for each individual sequence and its reconstruction was calculated, and the rounded up maximum loss value of 0.02142 was selected as the evaluation threshold.

### 3.2. Evaluation on the Left Bearing Unit

As an important remark, every subsequent model evaluation process is implemented on the filtered, normalized and sequential data values, the transformation of which is presented in Section 2.3.4, and the final dataset dimensionality is summarized in Table 4.

**Figure 10:**
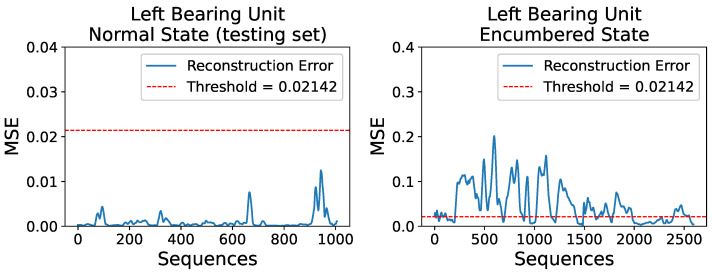
Reconstruction errors, in terms of MSE, for the left unit’s normal operational state testing set (**left**) and encumbered state set (**right**), compared to a set threshold of 0.02142 .

The performance of the AE model was initially evaluated on the reserved testing dataset of the left mounted ball bearing unit’s normal operational state, as it is segmented and displayed in Figure 8, for the purpose of confirming its generalization ability to data of the same behavior, which the model did not see during its training. After the 1006 sequences of the dataset were processed by the trained AE model and their reconstructions were obtained, the respective MSE losses were calculated and compared to the predetermined threshold of 0.02142 . An identical process was implemented for the examination of the left unit’s encumbered operational state and its dataset of 2595 sequences. Figure 10 displays the MSE loss values of the reconstructions of the two datasets mentioned.

As expected, newly introduced data of the same behavior as the training set are reconstructed adequately by the trained AE model and their MSE loss values are significantly below the selected threshold. Moreover, it is apparent that the majority of sequences on the left bearing unit’s encumbered state dataset have a considerably higher reconstruction error. Specifically, out of the 2595 sequences, 1739 are characterized as anomalous, while the rest are considered acceptable in terms of bearing operational behavior. Through several preliminary attempts, regarding model development and training, the accepted reconstructions of the encumbered state dataset amounted to over 2200 sequences, which is significantly higher compared to the current result; however, since the encumbered operational state was not erroneous to the industrial equipment, a less strict model was investigated and ultimately implemented.

**Figure 11:**
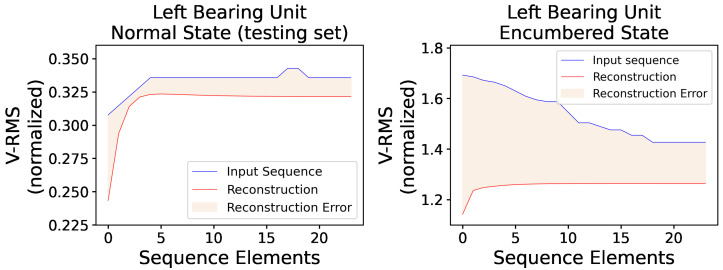
A random V-RMS sequence of the left mounted ball bearing unit’s normal operational state (**left**) and a random one of its encumbered operational state (**right**), along with their respective reconstructions as they were produced by the trained AE model, are displayed. The input sequences consist of 24 consecutive and normalized values, as obtained during the described data preprocessing.

**Figure 12:**
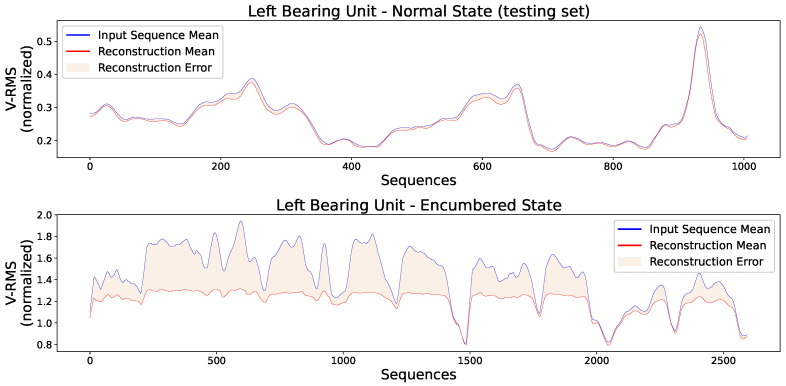
Each input point of the graphs represents the mean value of every original input sequence, while each reconstruction point represents the mean value of every respective outputted reconstruction sequence by the trained AE model. The V-RMS mean sequence values of the left bearing unit during its normal (**top**) and encumbered (**bottom**) operational state are presented, and all sequence means displayed are obtained through the normalized values.

**Figure 13:**
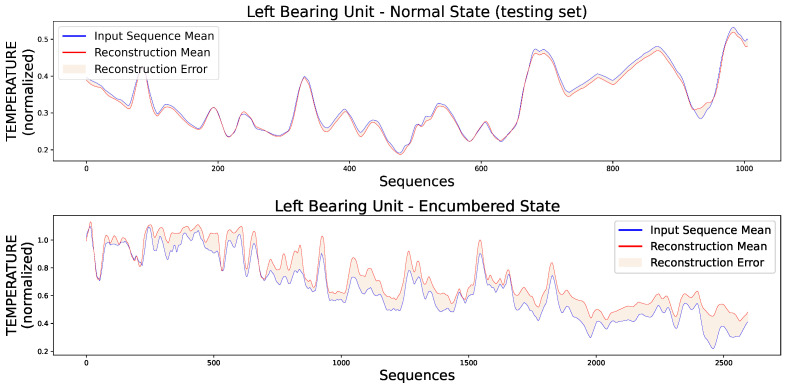
Each input point of the graphs represents the mean value of every original input sequence, while each reconstruction point represents the mean value of every respective outputted reconstruction sequence by the trained AE model. The Temperature mean sequence values of the left bearing unit during its normal (**top**) and encumbered (**bottom**) operational state are presented, and all sequence means displayed are obtained through the normalized values.

Additionally, Figure 11 presents two random original input sequences of normal and encumbered V-RMS values along with their reconstructions produced by the trained AE model, while Figure 12 and Figure 13 show the mean of each original and reconstructed sequence for the purpose of a visual overview of the entire time series of both V-RMS and Temperature attributes for the normal and encumbered operational states.

### 3.3. Evaluation on the Right Bearing Unit

**Figure 14:**
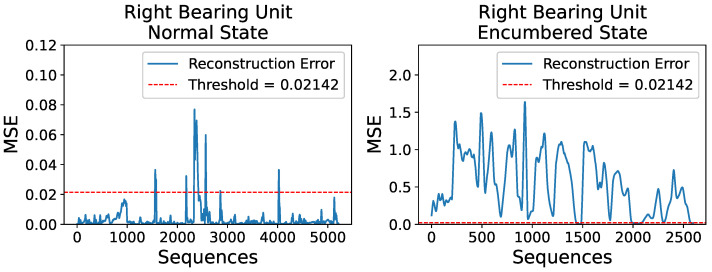
Reconstruction errors, in terms of MSE, for the right unit’s normal operational state set (**left**) and encumbered operational state set (**right**) compared to a set threshold of 0.02142 .

Using an identical procedure of model inference and MSE calculation, Figure 14 shows the respective reconstruction errors of the right bearing unit’s normal and encumbered operational behaviors.

The trained AE model clearly captured the slightly burdened circumstance halfway through the right unit’s normal operating condition, which was a fairly probable outcome, as well as two other occurrences before and after the obvious one mentioned, amounting to 165 anomalous sequences out of 5214. The two unexpected occurrences constitute as false positive results in terms of anomaly detection when, in fact, there is none since the data examined are assumed to be indicative of a normal operational behavior, although in the frames of an actual industrial production, a flagged anomaly would warrant a maintenance inspection. Furthermore, during the model’s evaluation on the encumbered operational state, only 165 of the 2595 sequences of the dataset were considered of acceptable operating condition, likely due to the right unit’s proximity to the centrifugal fan of the industrial blower which highly affected its readings.

**Figure 15:**
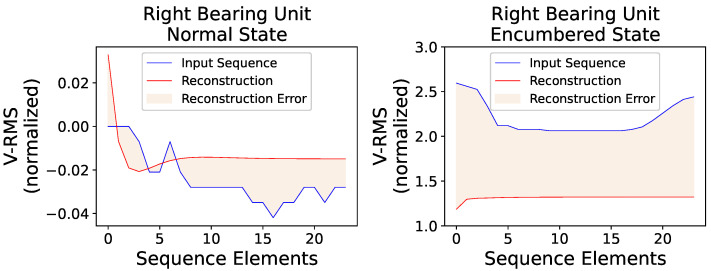
A random V-RMS sequence of the right mounted ball bearing unit’s normal operational state (**left**) and a random one of its encumbered operational state (**right**), along with their respective reconstructions, as they were produced by the trained AE model, are displayed. The input sequences consist of 24 consecutive and normalized values as obtained during the described data preprocessing.

**Figure 16:**
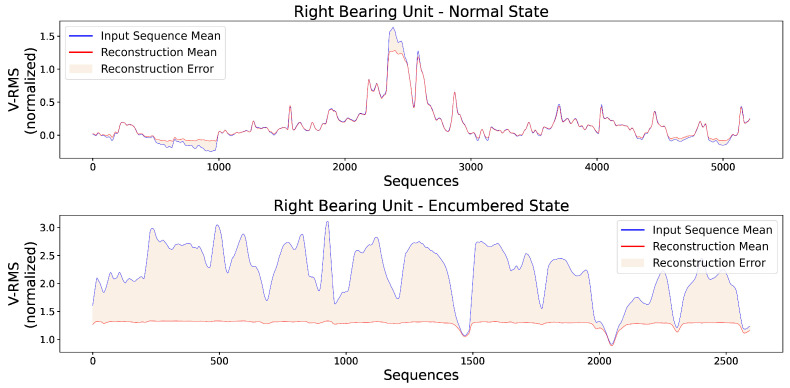
Each input point of the graphs represents the mean value of every original input sequence, while each reconstruction point represents the mean value of every respective outputted reconstruction sequence by the trained AE model. The V-RMS mean sequence values of the right bearing unit during its normal (**top**) and encumbered (**bottom**) operational state are presented, and all sequence means displayed are obtained through the normalized values.

**Figure 17:**
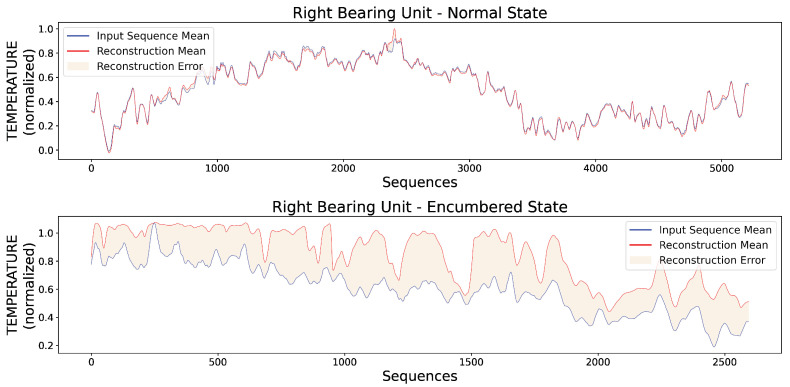
Each input point of the graphs represents the mean value of every original input sequence, while each reconstruction point represents the mean value of every respective outputted reconstruction sequence by the trained AE model. The Temperature mean sequence values of the right bearing unit during its normal (**top**) and encumbered (**bottom**) operational state are presented, and all sequence means displayed are obtained through the normalized values.

Similar to the visual overview of the previous subsection for the left bearing unit, Figure 15 shows two random VRMS sequences and their reconstructions for the right unit’s normal and encumbered state, while Figure 16 and Figure 17 present an average overview of the input and reconstructed sequences for the V-RMS and Temperature time series of the normal and encumbered operational states.

Finally, Table 5 offers a comparative overview of the Reconstruction Errors between normal and encumbered ball bearing unit operation that are presented in the previous Figures of Section 3, mainly Figure 11, Figure 12 and Figure 13 of the left bearing unit and Figure 15, Figure 16 and Figure 17 of the right bearing unit.

## 4. Discussion

To maintain their edge in their fields, businesses must strive for productivity and efficiency, thus more and more industries are taking advantage of I4.0 innovations by integrating them to their maintenance policies since healthy machinery and equipment failure mitigation allow sustainability as well as minimization of production costs.

In the present work, a sequence-to-sequence sparse stacked Autoencoder model was developed based on LSTM layers for the purpose of detecting anomalous behavior by processing consecutive multivariate sensor readings captured throughout production. The industrial machinery investigated was an industrial blower with sensor devices attached to its right and left mounted ball bearing units during normal and encumbered operational states. The available data were appropriately preprocessed and the model was trained on the left unit’s normal operation data before evaluated against the rest.

The trained AE model was successful in confirming the normal operational behavior, especially in the case of the right bearing unit, thus validating the model’s adaptability to similar equipment readings of the same machinery. Furthermore, the model was able to detect anomalous sequences in the burdened working condition on both bearing units despite deeming sequences of the encumbered dataset as acceptable due to its less strict nature. This was an acceptable outcome since the burdened working state of the actual machine was not necessary erroneous enough to cause a halt in production.

It should be noted that this research was made possible with the cooperation of industrial partners and is a part of larger project consisting of modules including data acquisition, real-time processing, visualization and alerting with the ultimate goal of transitioning from preventive to predictive maintenance policies. During the project, we had to overcome several implementation constraints, not only from a technical and scientific point of view but also practical difficulties concerning the uninterrupted operation routine of the production line. As a result, certain design decisions were dictated by the need of following non-invasive procedures in order to avoid disruption of the operation schedule of the plant.

Future work could include the development of a more complex, in terms of its architectural layers and number of nodes, AE model, or a stricter one regarding its regularization coding penalty. Application of these two future models using the current dataset can be in turn evaluated and compared to with the current implementation.

## 5. Conclusions

In conclusion, this study presented a deep learning approach for predictive maintenance of machinery using LSTM Autoencoders. The approach was applied to assess the operational behavior of two bearing units. A stacked sparse LSTM Autoencoder was trained on data from the left unit under normal operating conditions, allowing the model to learn the underlying patterns and statistical relationships within the data. The model’s performance was evaluated using the Mean Squared Error on data from the left unit in an encumbered state and on data from the right unit. The results showed that the model performed well on all datasets, demonstrating its ability to generalize and adapt to assessing the behavior of similar equipment. This approach has the potential to improve predictive maintenance practices by providing accurate and reliable assessments of machinery behavior.

The proposed prototype was implemented in Python and trained on custom real-world data. The results of the case study showed the potential for reducing redundant and preventive stoppages in the production line, thereby decreasing the cost of maintenance operations. However, further testing with larger datasets of high-quality data over a longer period is necessary to fully evaluate the performance and accuracy of the prototype. However, a serious difficulty remains the need for maintenance records to label the datasets with maintenance events such as component breakdowns, which may not be publicly available.

## Figures and Tables

**Table 1:** Implemented Framework.

Software	Version	Objective
Python	3.10.11	Main Programming Language
Numpy	1.22.4	Array Computations
Pandas	1.5.3	Data Analysis
Matplotlib	3.7.1	Graph Visualization
Scikit-learn	1.2.2	Preprocessing
Keras	2.12.0	Model Training

**Table 2:** Mean values of V-RMS and Temperature attributes.

	Standard Operation	Encumbered Operation	
V-RMS of left unit	0.002568	0.004556	m/s
V-RMS of right unit	0.002339	0.005906	
Temperature of left unit	37.93	43.58	Celsius
Temperature of right unit	37.15	41.80	

**Table 3:** Proposed Model Architecture.

	Input	Output
**Encoder**		
LSTM	24×2	24×8
LSTM	24×8	24×4
LSTM *	24×4	1×2
**Decoder**		
RepeatVector	1×2	24×2
LSTM	24×2	24×4
LSTM	24×4	24×8
TimeDistributed (Dense)	24×8	24×2

* L1 regularization penalty of 0.00015 applied to layer’s output.

**Table 4:** Dataset Dimensionality.

Dataset	Original Dimension *	Sequential Dimension
Left—Normal State (training set)	$\left(4185,2\right)$	$\left(4162,24,2\right)$
Left—Normal State (testing set)	$\left(1029,2\right)$	$\left(1006,24,2\right)$
Left—Encumbered State	$\left(2618,2\right)$	$\left(2595,24,2\right)$
Right—Normal State	$\left(5237,2\right)$	$\left(5214,24,2\right)$
Right—Encumbered State	$\left(2618,2\right)$	$\left(2595,24,2\right)$

* after median filtering.

**Table 5:** Reconstruction Errors (MSE).

Reconstructed Sample	Normal State	Encumbered State
Random V-RMS sequence of Left Bearing Unit	0.0003695	0.0897462
Mean V-RMS time series of Left Bearing Unit	0.0000993	0.0761422
Mean TEMPERATURE time series of Left Bearing Unit	0.0000795	0.0096677
Random V-RMS sequence of Right Bearing Unit	0.0002662	0.8246717
Mean V-RMS time series of Right Bearing Unit	0.0027108	1.0057427
Mean TEMPERATURE time series of Right Bearing Unit	0.0002835	0.0652557

## Data Availability

The data presented in this study are openly available in the Zenodo repository at https://doi.org/10.5281/zenodo.7994124, accessed on 1 June 2023.
